# *Staphylococcus lugdunensis* Endocarditis Complicated by Embolism in an 18-Year-Old Woman with Mitral Valve Prolapse

**DOI:** 10.1155/2013/730924

**Published:** 2013-01-29

**Authors:** Rosaria Pecoraro, Antonino Tuttolomondo, Gaspare Parrinello, Antonio Pinto, Giuseppe Licata

**Affiliations:** Dipartimento Biomedico di Medicina Interna e Specialistica, Università degli Studi di Palermo, 90127 Palermo, Italy

## Abstract

*Staphylococcus lugdunensis* is a coagulase-negative *staphylococcus* (CNS). It is a major cause of prosthetic valve endocarditis; mitral valve prolapse (MVP) has emerged as a prominent predisposing structural cardiac abnormality. We describe a case of *Staphylococcus lugdunensis* endocarditis in an 18-year-old woman with preexisting mitral valve prolapse complaining of fever, a one-month history of continuous-remittent fever (*T*_max_ 38.6°C). The transthoracic echocardiogram revealed large vegetation on the anterior mitral valve leaflet flopping from the atrial side to the ventricular side. Five sets of blood cultures were positive for coagulase-negative staphylococci. During hospitalization, after two weeks of antibiotic therapy, the patient complained of sudden pain in her right leg associated with numbness. Lower limb arterial Doppler ultrasound showed an arterial thrombosis of right common iliac artery. Transfemoral iliac embolectomy was promptly performed and on septic embolus *S. lugdunensis* with the same antibiotic sensitivity and the same MIC values was again isolated. Our patient underwent cardiac surgery: triangular resection of the A2 with removal of infected tissue including vegetation. Our case is an example of infective endocarditis by *S. lugdunensis* on native mitral valve in a young woman of 18 with anamnesis valve prolapse.

## 1. Introduction 

*Staphylococcus lugdunensis* is a coagulase-negative staphylococcus (CNS). It is a major cause of prosthetic valve endocarditis, particularly during the initial years after valve surgery, an important cause of nosocomial infective endocarditis (IE) and the cause of 3 to 8 percent of native valve endocarditis, usually in the setting of prior valve abnormalities; among these, mitral valve prolapse (MVP) has emerged as a prominent predisposing structural cardiac abnormality [1]. 

*S. lugdunensis*, which was first described in 1988 [2], was distinguished from other coagulase-negative staphylococcal species via DNA relatedness studies based on 11 clinical strains. Like other CNS, *S. lugdunensis* in humans ranges from a harmless skin commensal to a life-threatening pathogen (as with infective endocarditis) but it is considered unique among CNS because of its propensity for causing aggressive native valve infective endocarditis (IE) often fatal and usually community acquired [3].

 We describe a case of *Staphylococcus lugdunensis* endocarditis in an 18-year-old woman with preexisting mitral valve prolapse in order to emphasize the aggressiveness, as documented in the literature yet available, of this staphylococcus which takes on virulence characteristics similar to *Staphylococcus aureus.*

## 2. Case Report

### 2.1. History and Clinical Examination

An 18-year-old woman was sent to our ward from the emergency room (ER) complaining of fever, a one month history of continuous-remittent fever (*T*_max⁡_ 38.6°C) and musculoskeletal symptoms including arthralgias, myalgias, and a clear arthritis of ankle joint with signs of inflammation. She also complained of recurrent episodes of right eye superior quadrantanopia. Her past medical history included a mitral valve prolapse.

On admission physical examination of the patient was febrile with a body temperature of 37.9°C, her blood pressure was 105/80 mmHg, with a pulse rate of 108 beats/min, respiratory rate was 16/min, and oxygen saturation was 98.8%. On cardiac auscultation a midsystolic click a holosystolic murmur grade 3/6 with a maximum at the apex and radiating to the axillawas heard. There were no signs of heart failure. Examination of the respiratory system and the abdomen was normal, and there were no neurological abnormalities

There were no peripheral stigmata of endocarditis: no evidence of splinter or subungual hemorrhages, of Osler nodes and petechiae, and no hepatosplenomegaly. 

#### 2.1.1. Laboratory Tests and Investigations: From the ER to the Ward

The patient was sent to cardiology consultation from the ER and echocardiogram was performed, which showed thickened mitral leaflets and confirmed a mild mitral valve prolapse with mild regurgitation on Doppler. 

On admission hematological variables were abnormal showing anemia (hemoglobin level was 10.3 g/dL with normal chromic normal cystic red blood cell indices), a low serum iron level and high serum ferritin level, and leukocytosis with increased neutrophil count. Kidney function variables were normal, and serum troponin T was negative. Total bilirubin was 0.6 mg/dL, and transaminases were also normal. Her erythrocyte sedimentation rate (ESR) was 33 mm in the first hour, the C-reactive protein was elevated (CRP) (14 mg/dL), circulating immune complexes (CIC) were also assayed but these were normal, whereas rheumatoid factor and gamma-immunoglobulins were elevated. The urinalysis revealed proteinuria and microscopic hematuria. In view of anamnesis mitral valve prolapse and the detection of heart systolic murmur and fever, repeated blood cultures were taken, and transthoracic echocardiogram was repeated. 

**Figure 1:**
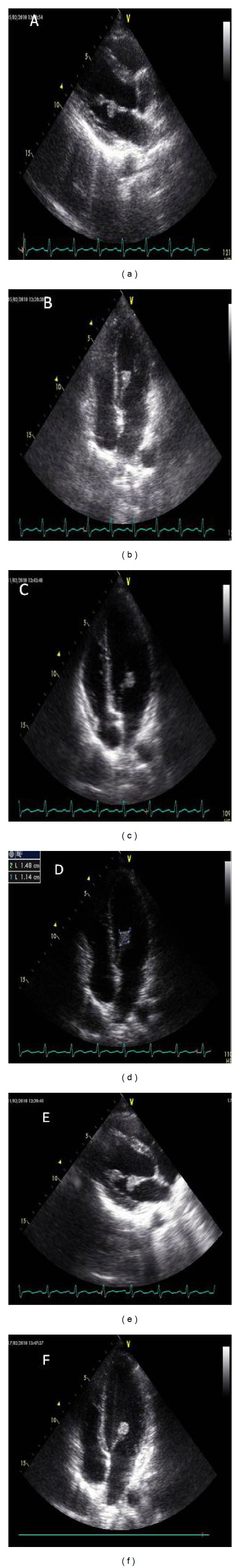
Echocardiographic findings in patients with *Staphylococcus lugdunensis *endocarditis.

The transthoracic echocardiogram revealed a large vegetation on the anterior mitral valve leaflet flopping from the atrial side to the ventricular side. The vegetation measured 1.03 × 1.66 cm. The patient had moderate mitral regurgitation (see Figure 1).

Pending the report of blood cultures the patient was initially treated empirically with intravenous administration of some antibiotics. 

Five sets of blood cultures were positive for coagulase-negative staphylococci. It was negative on tube coagulase testing but pyrrolidonyl arylamidase, ornithine decarboxylase, and fermentation positive, so *Staphylococcus lugdunensis* was definitively identified by the API ID32 staphylococcus, bioMérieux, Marcy l'Etoile. Susceptibility to antimicrobial agents was determined by a disk diffusion method and confirmed by a broth microdilution method, and organism was susceptible to several antibiotics (ciprofloxacin, levofloxacin, erythromycin, vancomycin, penicillin G, trimethoprim-sulfamethoxazole, daptomycin, gentamicin, and clindamycin). On the basis of positive blood cultures (>2) and echocardiograph finding, according to the modified Duke criteria [4], it was possible to diagnose acute infective endocarditis, and therefore the antibiotic therapy was changed; the patient began therapy with oxacillin (3 g every 6 h iv) + gentamicin (3 mg/kg for 24 h in three doses every 8 hours) as in the latest indication of the scientific literature in the treatment of endocarditis caused by staphylococci in native valves [5, 6]. The patient remained under observation.

### 2.2. Progression on Ward

A few days later no improvement was observed (patient still had a fever and the inflammatory markers persisted although slightly reduced: CRP: 7 mg/dL; GB: 12.200 N: 77% 1; ESR: 29 mm/1 h), so a new echocardiograph assessment was performed which showed unchanged vegetation size and a progression from mild to moderate mitral regurgitation at Doppler. 

In relation to both the evidence in the scientific literature of the high risk cardioembolic and destructiveness of endocarditis due to this causative agent and with our diagnostic hypothesis that anamnesis episodes of quadrantanopia were clinical manifestations suggestive of retinal embolism, heart surgery counseling was required, but it did not pose an indication for eventual surgery so the antibiotic therapy already set was continued.

Blood cultures, undertaken in the following days, confirmed the persistent state of sepsis by *Staphylococcus lugdunensis* (with the same antibiotic sensitivity profile); laboratory parameters showed no improvement. 

**Figure 2:**
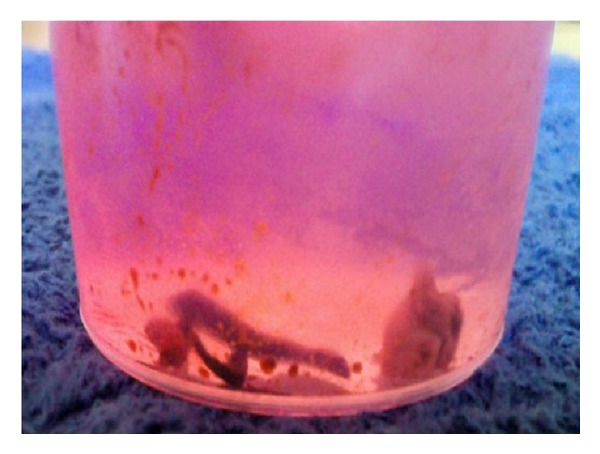
material obtained by embolectomy.

During hospitalization, after two weeks of antibiotic therapy, the patient complained of sudden pain in her right leg associated with numbness; at observation of the lower limb (foot and leg to the upper third) pale skin color with marble foot were observed, absence of posterior tibial and dorsalis pedis arterial pulse, and cool to the touch. So the patient underwent urgent lower limb arterial Doppler ultrasound which showed demodulation flow at the origin of the right common iliac artery, suggestive of total arterial thrombosis. Transfemoral iliac embolectomy was promptly performed and on septic embolus (see Figure 2) *S. lugdunensis*, with the same antibiotic sensitivity and the same MIC values, was again isolated.

The clinical picture required a new heart surgery consultation, and emergency surgery was performed. The patient underwent cardiac surgery: triangular resection of the A2 with removal of infected tissue including vegetation, reconstruction of the valve leaflet, and then positioning a pair of Gore-Tex artificial chords to support A2; posterior annuloplasty by implantation of Duran band 25 mm was also performed. 

In the postoperative heart surgery the patient has followed an intensive program of physical and respiratory rehabilitation with excellent recovery. At one-year follow-up our patient is well.

### 2.3. Discussion

*S. lugdunensis* is, commonly, a constituent of the human normal skin flora, preferentially colonizing the perineal region, and an infrequent, but not rare, human pathogen, and a rare contaminant in culture. Although CNS were historically considered innocuous or, rarely, opportunistic pathogens of low virulence, which have a major role in nosocomial infections [7], *S. lugdunensis* behaves more like *S. aureus,* exhibiting an elevated degree of virulence [8, 9]. 

The organism occasionally causes serious invasive infections such as osteomyelitis, septic arthritis in a native joint, prosthetic joint infections, spondylodiscitis, cellulitis, urinary tract infections, peritonitis, soft tissue abscesses or wounds, brain abscess, meningitis, ventriculitis, ventriculoperitoneal shunt infection, endometritis, infective endocarditis (*native valve endocarditis and prosthetic valve endocarditis*), myocarditis, pacemaker-related endocarditis, toxic shock syndrome, and septic shock [10, 11]. 

The majority of cases of IE caused by *S. lugdunensis* involve native valves, whereas other coagulase negative staphylococci (mainly *S. epidermidis*) infect the prosthetic valve. Mitral and aortic valve infection is often reported, and in some cases there is multivalvar endocarditis [12]. 

Most strains are penicillin and methicillin-susceptible but, despite the sensitivity of the organism in vitro, medical therapy is rarely successful, so most patients need surgery during the active phase of the disease. The first documented case of endocarditis caused by *S. lugdunensis* dates back to 1988, and not many case reports have since been published, almost all of which demonstrate the poor outcome of endocarditis caused by this aggressive organism except in cases of early heart surgery.

Liu et al. [11] have recently published a systematic Medline review (from January 1988 up to June 2008; 67 cases of *S. lugdunensis* IE) from which 67.2% cases of IE were community-acquired with an unknown portal of entry; 65.5% had comorbidities; native valve, prosthetic valve, and pacemaker IE accounted for 80.6%, 11.9%, and 7.5% of cases, respectively. Regarding antimicrobial susceptibility 82.0% of patients were susceptible to penicillin. About 87% of IE cases occurred on the left side. Of the left-side lesions, mitral valve, aortic valve, and both represented 40.3%, 32.8%, and 11.9% of the total cases. 11.9% cases had multi-valvular involvement, and all multivalvular lesions were on the left side. Eleven (16.4%) cases had the occurrence of embolization. Among patients that received echocardiography examinations, vegetation could be detected in 77.8% of cases. 68.7% of cases received both antimicrobial therapy and surgery. The overall mortality rate of *S. lugdunensis* IE was 38.8%. Yet on the basis of this paper the mortality rates for native valve endocarditis were 34.6, and upon univariate analysis, variables that had a significant association with mortality included age over 50 years, medical treatment alone, negative echocardiography findings and diagnosis before 1995. In the multivariate logistic regression analysis, medical treatment alone was an independent risk factor for mortality (odds ratio = 4.79; 95% confidence interval = 1.16–19.78).

Our case is an example of infective endocarditis by *Staphylococcus lugdunensis* on native mitral valve in a young woman of 18 with anamnesis valve prolapse. In the reported case the most likely hypothesis is that the infection was contracted during dental procedures [13], dating back to about fifteen days after onset of fever and skin lesion of the gluteal region. 

Consistent with the latest guidelines of the American College of Cardiology/American Heart Association, in this case targeted antimicrobial therapy [6] was used, but there was clinical and echocardiograph evidence already present which indicated surgery (anamnesis recurrent episodes of right eye amaurosis fugax and vegetation size). 

Our clinical case presented a number of points of interest:

- young age in the absence of comorbidity,
- originated on native valve with prolapse of the same
- uncertain origin (dental care? skin lesions?)
- possible retinal or brain embolization (explaining recurrent quadrantanopia) which would indicate urgent surgery in the beginning [14],
- systemic embolization (common iliac artery),
- delay in echocardiograph diagnosis,
- the need for surgical treatment despite the broad spectrum of microorganism antibiotic susceptibility (aggressive clinical course despite a non-MDR organism).
